# Lanthanum carbonate versus placebo for management of hyperphosphatemia in patients undergoing peritoneal dialysis: a subgroup analysis of a phase 2 randomized controlled study of dialysis patients

**DOI:** 10.1186/1471-2369-14-40

**Published:** 2013-02-18

**Authors:** Alastair J Hutchison, Maggie Gill, J Brian Copley, Lynne Poole, Rosamund J Wilson

**Affiliations:** 1 Manchester Institute of Nephrology and Transplantation, Manchester Royal Infirmary, Oxford Road, M13 9WL, Manchester, UK; 2 University of Manchester, Oxford Road, M13 9PL, Manchester, UK; 3 Shire Pharmaceuticals, Hampshire International Business Park, Chineham, RG24 8EP, Basingstoke, UK; 4 Shire Pharmaceuticals, 725 Chesterbrook Boulevard, 19087, Wayne, PA, USA; 5 Spica Consultants, Granary House, Granary Close, East Grafton, SW8 3UA, Marlborough, UK

**Keywords:** Chronic kidney disease, Lanthanum carbonate, Nutrition, Peritoneal dialysis, Phosphate control

## Abstract

**Background:**

This short-term study assessed the efficacy and safety of lanthanum carbonate in the treatment of hyperphosphatemia in dialysis patients; here, we report a prespecified subgroup analysis of patients undergoing peritoneal dialysis.

**Methods:**

Men and women (*n* = 39) who had received continuous ambulatory peritoneal dialysis for chronic kidney disease for 6 months or more were enrolled in eight renal medicine departments in the United Kingdom. A 2-week washout period was followed by a 4-week dose-titration phase during which patients received lanthanum carbonate titrated up to 2250 mg/day. This was followed by a 4-week, randomized, placebo-controlled, parallel-group phase during which patients continued to receive either lanthanum carbonate at the titrated dose, or a matched dose of placebo. The main outcome measure was control of serum phosphate levels (1.3-1.8 mmol/l) at the end of the parallel-group phase.

**Results:**

Serum phosphate was controlled in 3/39 (8%) patients at the beginning of the dose-titration phase (after washout) and in 18/31 (58%) patients treated with lanthanum carbonate at its end. After the parallel-group phase, 60% of lanthanum carbonate-treated patients and 10% of those receiving placebo had controlled serum phosphate. There was no difference in mean (95% confidence interval) serum phosphate levels between groups at randomization: lanthanum carbonate, 1.57 (1.34-1.81) mmol/l; placebo, 1.58 (1.40-1.76) mmol/l (p = 0.96). However, a difference was seen at the end of the parallel-group phase: lanthanum carbonate, 1.56 (1.33-1.79) mmol/l; placebo, 2.25 (1.81-2.68) mmol/l (p = 0.0015). There were no clinically important changes in nutritional parameters and no serious treatment-related adverse events were recorded.

**Conclusions:**

At doses up to 2250 mg/day, lanthanum carbonate is well tolerated and controls hyperphosphatemia effectively. Treatment with higher doses of lanthanum carbonate may allow patients undergoing peritoneal dialysis the potential to increase their dietary protein intake without compromising their phosphate control.

## Background

Up to 80% of patients with chronic kidney disease (CKD) stage 5 have levels of serum phosphate above the normal range (0.8-1.5 mmol/l) [1]. Hemodialysis or peritoneal dialysis are typically used as part of the management strategy in patients with CKD stage 5, but even with regular dialysis, serum phosphate often exceeds 2.1 mmol/l [2], a level associated with increased morbidity and mortality [3,4]. Restricting the intake of dietary phosphate to 800–1000 mg/day is recommended [5] to help manage hyperphosphatemia. However, care must be taken to maintain adequate protein intake because poor nutrition is also associated with increased mortality [6].

Recent reimbursement changes in the US healthcare system may result in greater use of peritoneal dialysis [7]. Malnutrition and comorbidities (including cardiovascular disease, chronic respiratory or liver disease, and carcinoma) are independently associated with mortality in patients undergoing peritoneal dialysis, and these two factors in combination are associated with the worst outcomes [8]. Protein loss across the peritoneal membrane during peritoneal dialysis places a greater nutritional burden on patients compared with those on hemodialysis, and this loss of protein is exacerbated by peritonitis, a major complication of peritoneal dialysis [9]. Maintaining adequate protein intake is therefore an important part of the management strategy for patients undergoing peritoneal dialysis. However, protein intake can be impeded by poor appetite, and increasing dietary protein can place an increased phosphate burden on the patient [10].

Poor appetite can be a side effect of peritoneal dialysis. Patients using a dialysate that contains dextrose, are exposed to an increased glucose load of 40–80 g/day [11]. This can result in both early satiety and gastroparesis [12], which, combined with compression of the stomach caused by the dialysate, lead to appetite suppression. Anorexia can also be caused by uremia, which can be a consequence of under dialysis [9]. Furthermore, patients undergoing peritoneal dialysis can develop dyslipidemia, because the high glucose load stimulates synthesis of hepatic lipoprotein [13] and increases insulin levels [14].

The importance of maintaining adequate dietary protein intake in patients undergoing peritoneal dialysis was highlighted in a recent study [15]. A daily protein intake of more than 0.94 g/kg/day was associated with the best outcomes in terms of both all-cause and cardiovascular mortality, and incidence of first-episode peritonitis, whereas an intake of less than 0.73 g/kg/day was associated with the worst outcomes. One way to manage the additional phosphate burden that results from increased protein intake [10] is concomitant use of a phosphate binder. Lanthanum carbonate is a highly effective phosphate binder [16] with demonstrated efficacy in patients receiving renal replacement therapy [17]; however, little has been published on its efficacy in managing hyperphosphatemia in patients undergoing peritoneal dialysis. Here, we report the results of a prespecified subgroup analysis of a previously published phase 2 study [18] in which the efficacy of lanthanum carbonate was assessed in patients undergoing continuous ambulatory peritoneal dialysis.

## Methods

### Patients

Men and women were eligible to enter the study if they were aged 18 years or over, had undergone continuous ambulatory peritoneal dialysis for 6 months or more, and had serum phosphate levels consistently above 1.8 mmol/l following washout of previous phosphate binders. Exclusion criteria included: patients with serum calcium levels above the upper limit of the normal range; severe hyperparathyroidism (parathyroid hormone [PTH] levels > 53 pmol/l (500 ng/l) [5]); serum phosphate levels > 3.0 mmol/l after the washout phase; other clinically significant abnormal laboratory values; a positive pregnancy test; significant gastrointestinal disorders (including known active peptic ulcer, Crohn’s disease, ulcerative colitis, irritable bowel syndrome, and past or present gastrointestinal malignancies); unstable dietary habits; life-threatening malignancy; or a positive status for human immunodeficiency virus. Patients with a history of drug or alcohol abuse, or who, in the opinion of the investigators, were unlikely to comply with treatment requirements, were also excluded. Patients were withdrawn if their serum phosphate levels exceeded 3.0 mmol/l, or if it was felt that continuation would be detrimental to them. The trial was conducted in accordance with the Guideline for Good Clinical Practice, and with the Declaration of Helsinki and its subsequent revisions. Ethical approval of the final protocol and its amendments was granted by the South and West Multicentre Research and Ethics Committee. Written informed consent was given by all patients.

### Study design

**Figure 1:**
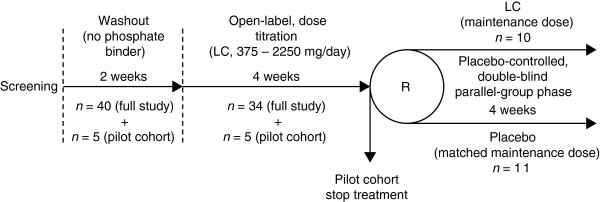
**Patient flow.** Dose-titration and parallel-group phases include patients in the safety set. LC: lanthanum carbonate; R: randomization.

This was a phase 2, multicenter, double-blind, placebo-controlled, parallel-group, dose-ranging study with a washout phase and two treatment phases (Figure 1). Patients did not receive any phosphate binder medication during the 2-week washout phase that followed screening. At the end of the washout phase, patients whose serum phosphate levels exceeded 1.8 mmol/l entered a 4-week, open-label phase, in which their dose of lanthanum carbonate (FOSRENOL®, Shire Pharmaceuticals, Nyon, Switzerland) was titrated to attain serum phosphate levels in the range 1.3-1.8 mmol/l. During this dose-titration phase, lanthanum carbonate doses were adjusted weekly according to patients’ serum phosphate levels, from an initial daily dose of 375 mg up to a maximum of 2250 mg.

A pilot cohort, consisting of five patients at one study center, was recruited to complete only the washout and dose-titration phases before patients were recruited into the full study. Data from the pilot cohort were reviewed to establish whether the proposed doses of lanthanum carbonate were consistent with acceptable safety and efficacy in patients with chronic renal failure prior to recruitment for the full study commencing at all centers.

On completion of the dose-titration phase, patients in the full study entered a 4-week, double-blind, placebo-controlled, parallel-group phase and were randomized in a 1:1 ratio to receive either their maintenance dose of lanthanum carbonate (as determined in the dose-titration phase) or a matched dose of placebo. There was no washout between the dose-titration and parallel-group phases.

Patients’ diets were monitored during the study using diet sheets and diary cards to confirm consistency of phosphate intake. Vitamin D was not initiated during the study, but continuation was allowed at an unchanged dose. Blood samples were collected at the start of the dose-titration phase and patients were assessed on a weekly basis throughout the study for changes in serum phosphate level. All patients received lanthanum carbonate as chewable tablets (125 or 250 mg), with the daily dose divided equally among three meals.

### Assessments

The primary efficacy endpoint, measured at the end of the parallel-group phase, was serum phosphate level in the range 1.3-1.8 mmol/l. Secondary endpoints included biochemical and hematological parameters and adverse events. Body weight, serum protein and serum albumin, are presented as measures of nutritional status. Weekly pill counts were performed to assess compliance to study medication.

### Analysis sets

Data were analyzed from patients who commenced dose titration (the safety set, including patients from the pilot cohort), all patients who were randomized to receive lanthanum carbonate or placebo (the randomized set), or all patients who received at least one dose of study medication in the parallel-group phase and had no protocol violations (the per protocol set).

### Statistical analysis

Treatment groups were compared with respect to continuous data using analysis of variance, Student’s *t*-test or Wilcoxon rank-sum test, as appropriate. Frequency distributions were tested using chi-squared or Fisher’s exact tests. The final titrated dose was compared between groups using the Wilcoxon rank-sum test. Treatment groups were compared with respect to serum phosphate, calcium and PTH levels using analysis of covariance, with the value at the end of the dose-titration phase used as covariate. Data relating to safety and tolerability, such as laboratory measures and vital signs, were tabulated and listed. Data at the end of the parallel-group phase were summarized both for week 4 and ‘last visit’. If the week 4 visit was missed, data from the last visit were carried forward.

### Role of the funding organization

The analysis reported here and the study from which the data are derived, were funded by Shire Pharmaceuticals (Basingstoke, UK). Three of the authors are employees of Shire Pharmaceuticals, and approval for publication was obtained from Shire Pharmaceuticals before submission of this manuscript.

## Results

### Patients

Of the 45 patients undergoing continuous ambulatory peritoneal dialysis who were screened, six were excluded from entering the dose-titration phase because their serum phosphate levels were 1.8 mmol/l or less at the end of the washout phase; the remaining 39 patients entered the dose-titration phase and were included in the safety set. There were eight withdrawals during dose titration: three due to adverse events, two to adverse events and withdrawal requests made by the patients, one to protocol violation, one to a serum phosphate level > 3.0 mmol/l, and one to a serum PTH level > 53 pmol/l (500 ng/l). Of the 31 patients who completed the dose-titration phase, five were excluded from the parallel-group phase following participation in the pilot cohort, three were excluded because their serum phosphate was uncontrolled, and two were withdrawn because of protocol violations. The remaining 21 patients were randomized to enter the parallel-group phase (lanthanum carbonate, *n* = 10; placebo, *n* = 11). Baseline characteristics for patients in the two groups were similar and are shown for all patients in Table 1. One patient in the placebo group was excluded from the per protocol set because of a protocol violation (serum phosphate level > 1.8 mmol/l on entry to the dose-titration phase). In addition, one patient in the placebo group discontinued during the parallel-group phase because of an adverse event.

**Table 1:** Patient baseline demographics; safety set

	**Dose-titration phase (*n*= 39)**	**Parallel-group phase**	
		**Lanthanum carbonate (*n*= 10)**	**Placebo (*n*= 11)**
Sex, *n* (%)			
Male	30 (76.9)	6 (60.0)	8 (72.7)
Female	9 (23.1)	4 (40.0)	3 (27.3)
Ethnicity, *n* (%)			
Asian	1 (2.6)	0 (0.0)	0 (0.0)
Caucasian	38 (97.4)	10 (100.0)	11 (100.0)
Age, years^a^	53.4 (15.6)	51.5 (17.5)	54.4 (15.3)
Duration of renal disease, years^b^	5.0 (1.0-41.0)	4.0 (1.0-21.0)	9.0 (1.0-41.0)
Duration of dialysis, months^b^	17.0 (6.0-107.0)	11.0 (6.0-87.0)	13.0 (6.0-107.0)
Previous transplant, *n* (%)	8 (20.5)	2 (20.0)	3 (27.3)

^a^Mean (standard deviation).

^b^Median (minimum, maximum).

The level of compliance with study medication was very high; mean compliance during the parallel-group phase was at least 95% for patients treated with lanthanum carbonate and at least 94% for patients receiving placebo. There were no marked differences in compliance for any visit.

### Phosphate control

The final titrated doses of lanthanum carbonate for all patients are given in Table 2; there was no significant difference in the final titrated doses between the groups entering the parallel-group phase (p = 0.85). At the start of the dose-titration phase, three patients (8%) had controlled serum phosphate and by the end of this phase, serum phosphate was controlled in 18 patients (58%). At the start of the parallel-group phase, seven (70%) of the patients randomized to each treatment group (*n* = 10 in each group; per protocol set) had controlled serum phosphate. At the end of this phase, six patients (60%) had controlled serum phosphate in the lanthanum carbonate group, compared with one (10%) in the placebo group (p = 0.057).

**Table 2:** Dose of lanthanum carbonate at the end of the dose-titration phase; safety set

**Dose, mg/day**	**Dose-titration phase (*n*= 39)**	**Parallel-group phase**	
		**Lanthanum carbonate (*n*= 10)**	**Placebo (*n*= 11)**
375	5 (12.8)	1 (10.0)	0 (0.0)
750	11 (28.2)	3 (30.0)	4 (36.4)
1500	14 (35.9)	3 (30.0)	4 (36.4)
2250	9 (23.1)	3 (30.0)	3 (27.3)

Data are presented as *n* (%).

**Figure 2:**
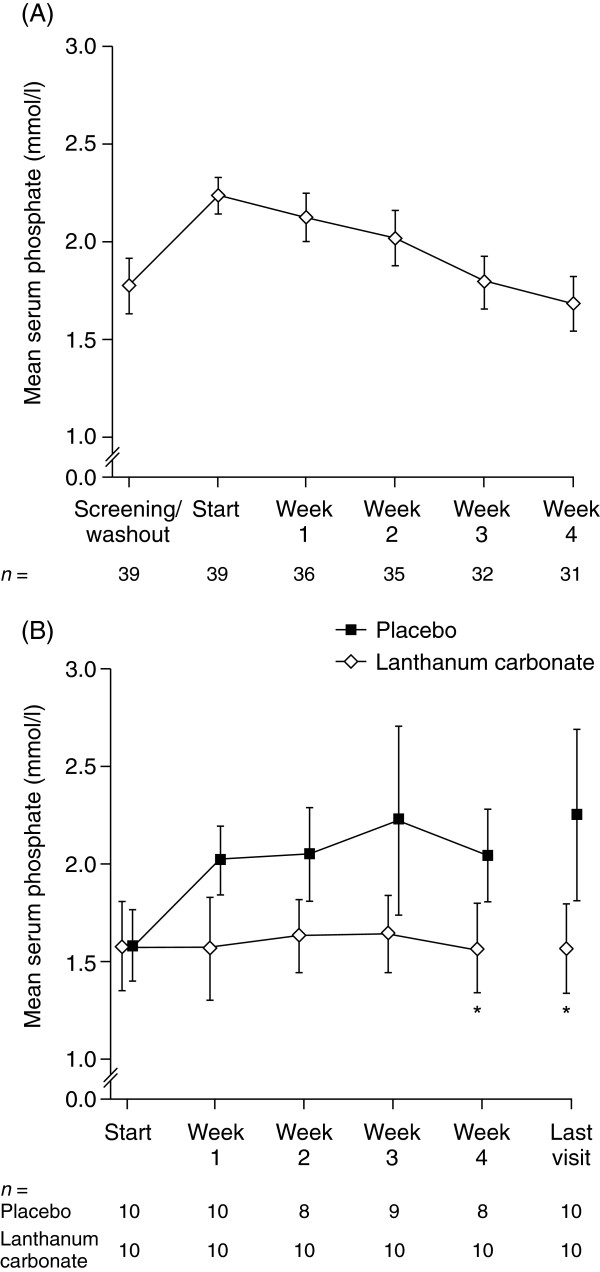
**Serum phosphate levels.** (**A**) during the open-label, lanthanum carbonate dose-titration phase (safety set); (**B**) during the randomized, placebo-controlled, parallel-group phase (per protocol set). ^*^p < 0.01 vs placebo. Data are presented as mean ± 95% confidence interval.

At the end of the dose-titration phase, the mean (95% confidence interval [CI]) serum phosphate level was 1.68 (1.54-1.81) mmol/l, compared with 2.23 (2.14-2.32) mmol/l at the start of the phase (Figure 2A). There was no significant difference in mean (95% CI) serum phosphate levels between the groups after randomization (the start of the parallel-group phase): lanthanum carbonate, 1.57 (1.34-1.81) mmol/l; placebo, 1.58 (1.40-1.76) mmol/l; (p = 0.96). After 1 week, the mean (95% CI) serum phosphate level in the placebo group was 2.02 (1.85-2.20) mmol/l and by the end of this phase the difference between the groups was significant: lanthanum carbonate, 1.56 (1.33-1.79) mmol/l; placebo, 2.25 (1.81-2.68) mmol/l; (p = 0.0015; Figure 2B).

### Nutritional parameters

Phosphate intake was derived from diary data, and the median intake was comparable between the two groups. At the end of the parallel-group phase, median daily phosphate intake was 34.75 mmol (1075.6 mg as phosphorus) in the lanthanum carbonate group and 32.57 mmol (1008.0 mg as phosphorus) in the placebo group. Furthermore, no clinically important changes in body weight or nutritional parameters were observed (Table 3). There was a small reduction in mean body weight, but mean serum albumin and total serum protein levels were stable during the dose-titration phase. During the parallel-group phase, the mean levels for body weight, total serum protein and serum albumin remained stable, or increased slightly in the lanthanum carbonate group, whereas all three were slightly reduced in the placebo group.

**Table 3:** Nutritional parameters; safety set

	**Dose-titration phase (*n*= 39)**	**Parallel-group phase**	
		**Lanthanum carbonate (*n*= 10)**	**Placebo (*n*= 11)**
Weight, kg			
Start of dose-titration phase	76.2 (14.1)	–	–
Start of parallel-group phase	74.9 (13.5)^a^	73.2 (16.0)	74.1 (12.0)
End of parallel-group phase	–	73.2 (17.0)	73.4 (13.6)^b^
Total serum protein, g/dl			
Start of dose-titration phase	6.9 (0.8)	6.7 (1.0)	6.9 (0.7)
Start of parallel-group phase	6.8 (0.7)^c^	6.6 (0.9)	6.9 (0.4)
End of parallel-group phase	–	6.9 (0.7)	6.7 (0.7)^d^
Serum albumin, g/dl			
Start of dose-titration phase	3.8 (0.4)	3.7 (0.4)	4.0 (0.3)
Start of parallel-group phase	3.7 (0.3)^c^	3.6 (0.4)	3.9 (0.3)
End of parallel-group phase	–	3.7 (0.4)	3.6 (0.4)^e^

Data are presented as mean (standard deviation).

^a^*n* = 25; ^b^*n* = 9; ^c^*n* = 31; ^d^*n* = 7; ^e^*n* = 8.

### Serum chemistry

There were no significant changes in mean serum calcium levels during the study. Mean (95% CI) levels of serum calcium were 2.32 (2.24-2.40) mmol/l on entry to the dose-titration phase and 2.38 (2.30-2.47) mmol/l at the end of this phase. There was no significant difference in mean serum calcium levels between the groups after randomization: lanthanum carbonate, 2.34 (2.18-2.49) mmol/l; placebo, 2.45 (2.23-2.68) mmol/l; (p = 0.350), and no significant difference between the groups at the end of the parallel-group phase: lanthanum carbonate, 2.36 (2.18-2.53) mmol/l; placebo, 2.42 (2.30-2.54) mmol/l; (p = 0.987). A small increase in mean serum PTH levels was seen in the placebo group during the parallel-group phase, but the difference between treatment groups was not statistically significant: mean (95% CI) change during the parallel-group phase: lanthanum carbonate, (0.39 (−3.00-3.78) pmol/l (3.67 [−28.27-35.61] ng/l); placebo, 4.57 (−0.72-9.86) pmol/l (43.11 [−6.74-92.96] ng/l); p = 0.148).

### Safety

There were no deaths or serious treatment-related adverse events during the study. Five patients were withdrawn during dose titration with lanthanum carbonate because of adverse events. Three of these were thought likely to be treatment-related (two with nausea and one with retrosternal pain), and two unlikely to be treatment-related (one with shoulder pain and one with menorrhagia). One patient was withdrawn while receiving placebo (anaphylactic shock following intraperitoneal treatment with vancomycin for peritonitis). During the dose-titration phase, 34 patients (87%) experienced at least one adverse event. In the parallel-group phase, seven patients (70%) in the lanthanum carbonate group and eight patients (73%) in the placebo group experienced at least one adverse event. The most commonly reported adverse events during dose titration included: vomiting, 10 (26%); nausea, 9 (23%); diarrhea, 3 (8%); and itching, 3 (8%). The adverse events most commonly reported by body system in both the lanthanum carbonate and placebo groups during the parallel-group phase were gastrointestinal (Table 4), and the only events reported by preferred term more than once in any body system were localized infection (2 [20%], lanthanum carbonate group) and coughing (2 [18%], placebo group).

**Table 4:** Adverse events; safety set

	**Dose-titration phase (*n*= 39)**	**Parallel-group phase**	
		**Lanthanum carbonate (*n*= 10)**	**Placebo (*n*= 11)**
Patients with any AE, *n* (%)	34 (87.2)	7 (70.0)	8 (72.7)
Total AEs, *n*	82	14	19
Patients withdrawing because of an AE, *n* (%)	5 (12.8)	–	1 (9.1)
Gastrointestinal AEs by preferred term, *n* (%)	20 (51.3)^a^	3 (30.0)^a^	3 (27.3)^a^
Abdominal distress	1 (2.6)	–	–
Bloating	2 (5.1)	–	–
Constipation	1 (2.6)	1 (10.0)	1 (9.1)
Dental disorder	–	1 (10.0)	–
Diarrhea	3 (7.7)	–	1 (9.1)
Flatulence	2 (5.1)	–	1 (9.1)
Indigestion	–	–	1 (9.1)
Mouth ulceration	1 (2.6)	–	–
Nausea	9 (23.1)	–	1 (9.1)
Peritonitis	2 (5.1)	–	–
Stools loose	–	1 (10.0)	–
Vomiting	10 (25.6)	1 (10.0)	1 (9.1)

^a^Some patients reported more than one gastrointestinal adverse event.

AE: adverse events.

## Discussion

This short-term clinical study demonstrates that lanthanum carbonate is an effective option for the control of serum phosphate in patients undergoing continuous ambulatory peritoneal dialysis. Of the 45 patients screened, six had a serum phosphate level of 1.8 mmol/l or less after washout and were thus excluded prior to dose titration. The use of food diaries was implemented during the washout phase so this may have reinforced the need for dietary control and led to a reduction in dietary phosphate intake in some patients. Following washout, 39 patients with uncontrolled serum phosphate (> 1.8 mmol/l) underwent dose titration with lanthanum carbonate, and after 4 weeks of treatment, the group mean serum phosphate level was 1.68 mmol/l, compared with a mean baseline value (after washout) of 2.23 mmol/l. During the parallel-group phase, the mean serum phosphate level in the placebo group was 2.02 mmol/l after 1 week, whereas serum phosphate levels remained stable in patients receiving lanthanum carbonate (Figure 2). After 4 weeks, the between-group difference in mean serum phosphate level was statistically significant (p = 0.0015). At the end of the study, 60% of patients had controlled serum phosphate in the lanthanum carbonate group compared with only 10% in the placebo group.

Serum phosphate levels can be managed in dialysis patients by restricting their intake of phosphate, but this may limit their intake of dietary protein, thereby compromising their nutritional status [6]. Maintenance of good nutritional status is important in terms of overall outcome for dialysis patients [7,15], and in those undergoing peritoneal dialysis there is an additional nutritional burden compared with hemodialysis patients, caused by serum protein loss across the peritoneal membrane. A small reduction in mean body weight that was recorded during the dose-titration phase of this study may have been caused by dietary adjustments made by patients trying to standardize their dietary phosphate intake in response to advice from dietitians. However, during the parallel-group phase, no change in mean body weight was seen in the lanthanum carbonate group, and other measures of nutritional status (mean serum albumin and total serum protein levels) were also unchanged. In comparison, mean body weight, serum albumin concentration and total serum protein concentration fell slightly in the placebo group during the parallel-group phase. Such changes may not be indicative of an overall trend given the short duration of treatment, but relatively small changes in serum albumin concentration over a sustained period can be associated with major changes in outcome. An increase of 0.3 g/dl or more over a 6-month period has been associated with a significant reduction in all-cause mortality in patients undergoing peritoneal dialysis, while a reduction of 0.2 g/dl or greater over the same period has been associated with a significant increase in mortality [19].

Lanthanum carbonate was well tolerated in this study; the majority of adverse events were gastrointestinal and occurred to a similar extent in both randomized groups. This is in contrast to a recent study in patients on peritoneal dialysis where 15/35 patients on lanthanum carbonate were withdrawn, 10 of whom had gastrointestinal complications [20]. Other studies have reported no withdrawals due to adverse events and variable numbers of mild or moderate events [21-23]. In the present study adverse events led 5/39 patients to be withdrawn from the dose-titration phase; three of these were thought likely to be treatment-related. Levels of serum markers relevant to CKD (such as calcium and PTH) were essentially unchanged throughout the study. The maximum daily dose of lanthanum carbonate used in this study was 2250 mg and most patients received 1500 mg/day or less. This is considerably lower than the daily dose of 3000 mg now used in many countries. At this higher dose, it would be anticipated that more dietary phosphate could be removed and, therefore, if patients were to receive lanthanum carbonate at this dose they may have the potential to improve their nutritional status by increasing their dietary protein intake without compromising serum phosphate control. While not clinically important, the mean levels of serum albumin observed (as a marker of nutritional status) were slightly below the level of 4 g/dl that is recommended in maintenance dialysis patients [24].

Increasing the dose of a medication is usually associated with an increase in tablet burden, a factor that is an important consideration in a patient group in which therapeutic regimens are demanding. However, lanthanum carbonate can be given at a dose of 3000 mg/day in three tablets, so the daily dose can be increased to this level without increasing tablet burden. A recent study found that when patients switched from another phosphate binder to lanthanum carbonate, their daily tablet burden was reduced by approximately 2–8 tablets [25].

Oral phosphate binders alone are unlikely to fully control serum phosphate levels, and must be administered in conjunction with a careful review of peritoneal dialysis adequacy and prescription, as well as dietetic review and counselling to ensure that dietary intake is controlled. These three measures taken together will provide optimal phosphate control in the majority of cases.

Potential limitations of this study, relating to possible bias introduced by exclusion criteria and the protocol for withdrawals from the trial, were considered and placed in context in a previous publication [18]. Additional limitations include the short duration of the study and the small number of patients following randomization. However, recent longer-term (8–48 week) studies have demonstrated that lanthanum carbonate has a phosphate-lowering effect on patients on peritoneal dialysis [21-23]. A further limitation is that dialysis adequacy was not assessed throughout the study, and any between-group differences in this parameter could contribute to altered phosphate levels.

It is also worth noting that dietary data were collected in this study primarily for the purpose of ensuring consistency of dietary phosphate intake. Given the importance of maintaining adequate nutritional status in patients undergoing peritoneal dialysis, this trial would have benefited from more extensive data collection on calorie and protein intake. Given the promising data presented here on the level of serum phosphate control achievable with a relatively modest dose of lanthanum carbonate in patients on peritoneal dialysis, a longer and more detailed investigation into the nutritional status of such patients receiving higher doses of lanthanum carbonate may be warranted.

## Conclusion

At doses up to 2250 mg/day, lanthanum carbonate is well tolerated and controls hyperphosphatemia effectively. Treatment with higher doses of lanthanum carbonate may allow patients undergoing peritoneal dialysis the potential to increase their dietary protein intake without compromising their phosphate control.

## Abbreviations

CI: Confidence interval;CKD: Chronic kidney disease;PTH: Parathyroid hormone

## Competing interests

AH has received research funding and honoraria from Shire Pharmaceuticals for various activities. MG, JBC and LP are employees of Shire Pharmaceuticals. RW is a consultant to Shire Pharmaceuticals.

## Authors’ contributions

AH was the principal investigator, was involved in the design and conduct of the study, collection and interpretation of the data and writing and review of the manuscript. MG was involved in interpretation of the data and writing and review of the manuscript. JBC was involved in interpretation of the data and writing and review of the manuscript. LP was involved in analysis and interpretation of the data and writing and review of the manuscript. RW was involved in analysis and interpretation of the data and writing and review of the manuscript. All authors read and approved the final manuscript.

## Pre-publication history

The pre-publication history for this paper can be accessed here:

http://www.biomedcentral.com/1471-2369/14/40/prepub

## References

[B1] Block GA Hulbert-Shearon TE Levin NW Port FK Association of serum phosphorus and calcium x phosphate product with mortality risk in chronic hemodialysis patients: a national study Am J Kidney Dis 1998 31 607 617 10.1053/ajkd.1998.v31.pm9531176 9531176

[B2] Block GA Port FK Re-evaluation of risks associated with hyperphosphatemia and hyperparathyroidism in dialysis patients: recommendations for a change in management Am J Kidney Dis 2000 35 1226 1237 10.1016/S0272-6386(00)70064-3 10845841

[B3] Kestenbaum B Sampson JN Rudser KD Patterson DJ Seliger SL Young B Sherrard DJ Andress DL Serum phosphate levels and mortality risk among people with chronic kidney disease J Am Soc Nephrol 2005 16 520 528 10.1681/ASN.2004070602 15615819

[B4] Block GA Klassen PS Lazarus JM Ofsthun N Lowrie EG Chertow GM Mineral metabolism, mortality, and morbidity in maintenance hemodialysis J Am Soc Nephrol 2004 15 2208 2218 10.1097/01.ASN.0000133041.27682.A2 15284307

[B5] Eknoyan G Levin A Levin N National kidney foundation. K/DOQI clinical practice guidelines for bone metabolism and disease in chronic kidney disease Am J Kidney Dis 2003 42 Suppl 3 S1 S201 14520607

[B6] Shinaberger CS Greenland S Kopple JD Van Wyck D Mehrotra R Kovesdy CP Kalantar-Zadeh K Is controlling phosphorus by decreasing dietary protein intake beneficial or harmful in persons with chronic kidney disease? Am J Clin Nutr 2008 88 1511 1518 10.3945/ajcn.2008.26665 19064510 PMC5500249

[B7] Golper TA Guest S Glickman JD Turk J Pulliam JP Home dialysis in the new USA bundled payment plan: implications and impact Perit Dial Int 2011 31 12 16 10.3747/pdi.2010.00143 21282384

[B8] Prasad N Gupta A Sinha A Sharma RK Saxena A Kaul A Bhaduria D Gupta A Confounding effect of comorbidities and malnutrition on survival of peritoneal dialysis patients J Ren Nutr 2010 20 384 391 10.1053/j.jrn.2010.01.001 20226687

[B9] Bergström J Fürst P Alvestrand A Lindholm B Protein and energy intake, nitrogen balance and nitrogen losses in patients treated with continuous ambulatory peritoneal dialysis Kidney Int 1993 44 1048 1057 10.1038/ki.1993.347 8264134

[B10] Boaz M Smetana S Regression equation predicts dietary phosphorus intake from estimate of dietary protein intake J Am Diet Assoc 1996 96 1268 1270 10.1016/S0002-8223(96)00331-8 8948388

[B11] Gallieni M Musetti C Granata A Olivi L Bertoli S Metabolic consequences of peritoneal dialysis treatment Panminerva Med 2009 51 175 185 19859052

[B12] Van Vlem BA Schoonjans RS Struijk DG Verbanck JJ Vanholder RC Van Biesen WV Lefebvre RA De Vos MP Lameire NH Influence of dialysate on gastric emptying time in peritoneal dialysis patients Perit Dial Int 2002 22 32 38 11929141

[B13] Johansson AC Samuelsson O Attman PO Haraldsson B Moberly J Knight-Gibson C Alaupovic P Dyslipidemia in peritoneal dialysis – relation to dialytic variables Perit Dial Int 2000 20 306 314 10898048

[B14] Lindholm B Norbeck HE Serum lipids and lipoproteins during continuous ambulatory peritoneal dialysis Acta Med Scand 1986 220 143 151 3776689 10.1111/j.0954-6820.1986.tb02742.x

[B15] Dong J Li Y Xu Y Xu R Daily protein intake and survival in patients on peritoneal dialysis Nephrol Dial Transplant 2011 26 3715 3721 10.1093/ndt/gfr142 21430179

[B16] Martin P Wang P Robinson A Poole L Dragone J Smyth M Pratt R Comparison of dietary phosphate absorption after single doses of lanthanum carbonate and sevelamer carbonate in healthy volunteers: a balance study Am J Kidney Dis 2011 57 700 706 10.1053/j.ajkd.2010.11.028 21354682

[B17] Hutchison AJ Barnett ME Krause R Kwan JTC Siami GA SPD405-309 lanthanum study group Long-term efficacy and safety profile of lanthanum carbonate: results for up to 6 years of treatment Nephron Clin Pract 2008 110 c15 c23 10.1159/000149239 18667837 PMC2790759

[B18] Al-Baaj F Speake M Hutchison AJ Control of serum phosphate by oral lanthanum carbonate in patients undergoing haemodialysis and continuous ambulatory peritoneal dialysis in a short-term, placebo-controlled study Nephrol Dial Transplant 2005 20 775 782 10.1093/ndt/gfh693 15703206

[B19] Mehrotra R Duong U Jiwakanon S Kovesdy CP Moran J Kopple JD Kalantar-Zadeh K Serum albumin as a predictor of mortality in peritoneal dialysis: comparisons with haemodialysis Am J Kidney Dis 2011 58 418 428 10.1053/j.ajkd.2011.03.018 21601335 PMC3159826

[B20] Lee YK Choi HY Shin SK Lee HY Effect of lanthanum carbonate on phosphate control in continuous ambulatory peritoneal dialysis patients in Korea: a randomized prospective study Clin Nephrol 2012 Dec 4 [Epub ahead of print] 10.5414/CN107362 23211335

[B21] Kawanishi H Ishida M Ishizaki M Takuma Y Tamura H Kobayashi S Tamura T Ohashi H Hiramatsu M Minakuchi J Hirakata H Shigematsu T Lanthanum carbonate treatment of patients with hyperphosphatemia undergoing CAPD Perit Dial Int 2008 28 673 675 18981401

[B22] Ohno M Ohashi H Oda H Yokoyama H Okada M Nagaya M Izumi K Ito H Katoh S Lanthanum carbonate for hyperphosphatemia in patients on peritoneal dialysis Perit Dial Int 2012 [Epub ahead of print] 10.3747/pdi.2012.00600 PMC3649899 23209037

[B23] Yamada S Yoshida H Taniguchi M Tanaka S Eriguchi M Nakano T Tsuruya K Kitazono T Effectiveness of lanthanum carbonate treatment used in combination with other phosphate binders in peritoneal dialysis patients Intern Med 2012 51 2097 2104 10.2169/internalmedicine.51.6814 22892485

[B24] Eknoyan G Levin N Clinical practice guidelines for nutrition in chronic renal failure K/DOQI, National kidney foundation Am J Kidney Dis 2000 35 S1 S14010895784

[B25] Vemuri N Michelis MF Matalon A Conversion to lanthanum carbonate monotherapy effectively controls serum phosphorus with a reduced tablet burden: a multicenter open-label study BMC Nephrol 2011 12 49 10.1186/1471-2369-12-49 21962172 PMC3197476

